# Authentic Leadership and Its Relationship With Job Satisfaction: The Mediator Role of Organizational Dehumanization

**DOI:** 10.5964/ejop.6125

**Published:** 2022-11-30

**Authors:** Matías Arriagada-Venegas, Eva Ariño-Mateo, Raúl Ramírez-Vielma, Gabriela Nazar-Carter, David Pérez-Jorge

**Affiliations:** 1 Universidad de Concepción, Concepción, Chile; 2 Universidad Europea de Valencia, València, Spain; 3 Universidad de La Laguna, San Cristóbal de La Laguna, Spain; University of Wollongong, Wollongong, Australia

**Keywords:** authentic leadership, job satisfaction, organizational dehumanization, emotions, mechanistic dehumanization

## Abstract

The objective of this research was to examine the mediating role that organizational dehumanization plays between authentic leadership and job satisfaction. The study was carried out with a sample of 422 participants, 50.7% were men and 49.3% women, with an average age of 38.96 years. The workers belong to different public and private organizations in Chile, and they responded to instruments of sociodemographic characterization, employment history and the scales of organizational dehumanization, authentic leadership, and job satisfaction. Data analysis included descriptive, correlational, and mediation analyses. The results allow us to maintain the hypothesis that organizational dehumanization plays a mediator role in the relationship between authentic leadership and job satisfaction. Implications of these findings are discussed.

In the 1936 film starring Charles Chaplin, *Modern Times*, the conditions of workers in the industry were satirically described. The worker has no name, is known as "the factory worker" and tightens bolts on an assembly line in a highly automatized factory. Like his co-workers, all his actions are repetitive and mechanical. Every human movement depends on the machine and the slightest distraction interrupts the entire production cycle. In one of the movie's most famous scenes, the worker moves slowly on the assembly line and is sucked under the gears of the machine, metaphorically becoming another part of the machine.

Similarly, various economists and philosophers have reflected on this phenomenon. Fromm (1974) states that workers become "specialized tools", which are judged based on their efficiency and productivity, while their human qualities are devalued. This perspective is called organizational dehumanization, and it is understood as the experience that employees have when they are objectified, their personal subjectivity is denied, and it is perceived as a tool or instrument for the purposes of the organization (Bell & Khoury, 2011). The most common form of dehumanization within an organization refers to both intentional mistreatment and abuse of workers, and involuntary disregard for their well-being (Bell & Khoury, 2016).

Over the last decades, the interest in studying the way in which workers perceive the relationship with their organization has grown, and one of the resulting lines of research has been dehumanization field applied to work environments (Bell & Khoury, 2011, 2016; Caesens et al., 2017; Christoff, 2014). Dehumanization has been described as a frequent experience (for workers) in modern organizational contexts (Christoff, 2014), but more research is needed on this phenomenon and its consequences at workers and the organizational level.

This research adds to the study of this phenomenon and seeks to increase the scarce knowledge of organizational dehumanization at the workplace and its relationship with other organizational variables, specifically authentic leadership and job satisfaction.

Authentic leadership is a type of worker-focused leadership (Walumbwa et al., 2008) that defends the ethical base of both leaders and organizations and there is evidence about its positive effects for workers and organizations (Gardner et al., 2005). Therefore, it is interesting to study how the lack of authentic leadership can generate higher levels of organizational dehumanization. In general, authentic leadership has been studied with positive variables in organizational environments (Valsania et al., 2012; Wang et al., 2021). Likewise, the variable job satisfaction was analysed, because occupies a central place in the experience of people at work and is related to performance and organizational strategies (Böckerman & Ilmakunnas, 2012).

## Theoretical Background and Hypotheses

### Dehumanization

The beginnings of the Theory of Dehumanization start with Kelman (1973) and Staub (1989) who examined this phenomenon from the context of mass violence. Likewise, Opotow (1990) analysed dehumanization as one of the several forms of moral exclusion in which people were placed out from where moral values, rules and considerations of justice are applied. From these authors, dehumanization has been analysed, from the Infrahumanization Theory, proposed by Leyens et al. (2001) and the Dual Model of Dehumanization of Haslam (2006).

The Infrahumanization Theory was born from a team of psychologists in Belgium and Spain who started the study of processes related to dehumanization from the anthropological assumptions that ethnic groups often reserve the "human essence" for themselves. Leyens et al. (2001) theorized that this form of ethnocentrism could be a generalized phenomenon and created the Theory of Infrahumanization stating that people tend to perceive the outgroup as less human than the ingroup or, which means, to attribute fewer secondary emotions to members of the outgroup compared to the ingroup. The different attributions of emotions distinguish between humans and animals, as animals can just perceive primary emotion as joy, but no secondary, as happiness, because it requires more cognitive development, characteristic of humans. Furthermore, Haslam (2006), took a step forward and proposed that human singularity, as the distinction between humans and animals is just one more way to dehumanize. The author adds that people can also dehumanize others by perceiving them as inanimate objects, such as robots or objects. Haslam has remarked two central aspects that define the human condition: exclusively human traits (Human Uniqueness; HU), which differentiate the exclusively human characteristics from animal´s (cognitive ability, civility and refinement), and human nature (Human Nature; HN), which integrates typical characteristics of human beings’ behaviour such as emotionality, vitality and warmth.

Exclusively human traits require greater cognitive maturity, appear later in time, and differ across cultures, while those that embody human nature are essential, universal, and emotionally related. These two sets of characteristics differentiate humans from animals and robots, respectively, and have been investigated in various contexts and cultures (Haslam et al., 2005; Haslam et al., 2008; Haslam & Loughnan, 2014).

From a labour point of view, dehumanization in organizations refers to the denial or diminished attribution of humanity to workers or leaders, who can be considered or metaphorized as objects. There are several studies that have analysed the consequences of dehumanization, to highlight Bell and Khoury (2011), who affirm that dehumanization is a negative experience that affects the individual and can cause dissociation from the organization. Likewise, Christoff (2014), states that dehumanization can harm the well-being of the workers since it increases the level of anxiety or depression and reduces the need for competition and interaction among the workers. Additionally, Baldissarri et al. (2014), found that workers who felt being perceived as an instrument by their supervisor reported higher levels of burnout. Organizational justice and intrinsic characteristics of work design such as repetition of movements, fragmentation of activities and dependency also promote the perception of organizational dehumanization in workers (Andrighetto et al., 2017; Bell & Khoury, 2016).

Dehumanization not only has negative consequences, but it can also have certain benefits. In the workplace, some subtle forms of dehumanization have been perceived positively, for example, managers and coordinators who act empathetically and coldly toward workers have been shown to make better decisions from the organization's point of view (Christoff, 2014). Furthermore, dehumanizing is considered beneficial for effective performance in the health sector. Specifically, it has been shown that when doctors need to inflict pain on their patients derived from some treatments or diagnostic tests, it is useful to dehumanize them, and in this way, a psychological distance is maintained (Haque & Waytz, 2012; Lammers & Stapel, 2011). Beyond the medical context, dehumanization has been shown to help people in positions of power to establish a personal distance from others and make rational decisions, even if they cause harm to other people (Christoff, 2014).

Dehumanization also has consequences for people who suffer from it. People who are mechanically dehumanized are considered objects, that is, beings lacking the ability to have emotions. These people tend to enter "cognitive deconstructive" states that are characterized by less clarity of thought, emotional numbness, and cognitive inflexibility. Being dehumanized leads to widespread emotions of sadness, anger, guilt, and shame (Bastian & Haslam, 2011). It has been shown that when a person is dehumanized, the status of this person is reduced and attitudes of condescension and degradation towards this person are maintained, as this person is perceived as incompetent and unsophisticated (Vohs et al., 2007). In this regard, Bastian and Haslam (2011) demonstrated that social ostracism increases the perception of mechanistic dehumanization, mainly because having no emotions leads to seeing oneself as an object, emotionally inert, cold and rigid, and to entering cognitive deconstructive states (Haslam et al., 2005; Twenge et al., 2003).

Another study indicates that there is a relationship between dehumanization and the organization's ethical climate. Within organizations, a certain ethical climate is generated, especially driven by management and leader styles, either by action or omission, and from this fact, workers can perceive types of abuse or dehumanizing behaviours (Wiener et al., 2014). Väyrynen and Laari-Salmela (2018), point out the importance of the ethical climate in the organization and affirm that when the organization does not show respect and dignity to its workers, they perceive that they cannot trust the organization. In this way, the perception of respect for humanity and dignity creates the necessary environment for trust to exist and for an ethical organizational climate to be produced, and all of this has consequences on the results of work, in terms of performance and quality of work, as well as the reduction of confidence (Väyrynen & Laari-Salmela, 2018) and the commitment of workers towards their leader or organization (Martin & Cullen, 2006).

Recently, Caesens et al. (2017), demonstrated that dehumanization plays a mediating role between the organization's perception of support and worker satisfaction.

### Authentic Leadership and Organizational Dehumanization

Authentic leadership dates back to the early 2000s along with Luthans and Avolio (2003) but took precedence in academia in the 2008 subprime crisis, which caused economic and social instability around the world. This scenario raised a question mark for the so-called “transformational” leaders, who, while showing positive results through behaviors based on charisma, inspiration and individual stimulation, act to achieve their personal interests. Instead, authentic leadership, apart from these characteristics, focuses on how ethics should shape the behaviour of leaders (Gil et al., 2011; Simola et al., 2010).

Authentic leadership is a multidimensional model (Walumbwa et al., 2008) that includes; (a) the leader's self-awareness (strengths, ways of improvement and own motivations of the leader; Kernis, 2003), (b) relational transparency (leaders openly share information and encourage their collaborators to do the same; Avolio & Gardner, 2005; Gardner et al., 2005), (c) internalized moral perspective (capacity regulation; Ryan & Deci, 2003) and (4) balanced processing (ability to objectively analyze information; Gardner et al., 2005). These dimensions were measured by the Authentic Leadership Questionnaire (ALQ; Walumbwa et al., 2008).

There is evidence of the positive influence of authentic leadership in the organization. Research has shown the benefits of authentic leadership in the processes of social identification of workers, specifically with their organization and with their workgroup (Gardner et al., 2005; Ilies et al., 2005; Wong et al., 2010). This is due to relational transparency, since relations with workers are based on sincerity and honesty and involves an active process of opening and developing intimacy and trust with followers (Goldman & Kernis, 2002). In this sense, Caesens et al. (2017) demonstrate that there is a relationship between the perception of support by the organization and the perception of dehumanization, and the fact that the worker does not perceive supported by his company is the preamble to organizational dehumanization. In the same way, Eisenberger et al. (2002) state that immediate supervisors influence the organization's perception of support in general, thus, leadership, and specifically authentic leadership, could explain the employee's perception of being excluded/included by the organization.

Furthermore, the experience or position of power has been shown to influence dehumanization, since powerful people tend to be psychologically more closed than others and maintain a greater interpersonal distance from others (Anderson et al., 2003; Lee & Tiedens, 2001). Instead, the authentic leader is committed to a closer and more trustworthy relationship with followers, which is why a decrease in the perceptions of being seen as an object by the worker could be expected (Lammers & Stapel, 2011; Walumbwa et al., 2008).

On the other hand, Renger et al. (2016) have stated that organizational justice, understood as those perceptions that workers have about what is fair in the organizational environment, can help employees to protect themselves from dehumanization. These authors demonstrated that a high (dis)respect towards the worker, understood as the (in) equality received towards the members of the group, enhances the perception of being treated as a (non) human being. This is because organizational justice is related to the need to be recognized as an individual who adds value to the organization or the work team. Recent studies have shown the positive relationship between the perception of organizational justice and authentic leadership, showing that organizational justice works as a mediating variable when analysed with group identification and commitment to the supervisor (Emuwa, 2013; García-Guiu et al., 2015).

A good leadership style and a pleasant work environment have been shown to help employees meet their fundamental needs, such as the need for belonging, relationship or respect (Bell & Khoury, 2016). Thus, given the above arguments, it is stated:

*H1*: Authentic leadership is negatively related to the perception of organizational dehumanization.

### Organizational Dehumanization and Job Satisfaction

Building on Haslam's (2006) mechanistic dehumanization, several researchers have suggested that the experience of perceiving dehumanized is detrimental to the psychological well-being of individuals (Bastian & Haslam, 2011; Christoff, 2014). Along the same lines, Shore and Coyle-Shapiro (2012), indicated that working in an organization that is destructive and degrading for workers increases their perception of relational devaluation, injustice, and frustration because of the impossibility of meeting their basic needs (self-esteem or belonging). In addition, it negatively influences the perception that the worker has of his work. Several investigations have shown that violation of basic needs has a detrimental effect on workers' health and subjective well-being (Gillet et al., 2012; Shore & Coyle-Shapiro, 2012). On the contrary, the fulfilment of basic needs is consistently associated with subjective well-being (Tay & Diener, 2011).

According to the Theory of Self-Determination (Ryan & Deci, 2000), psychological well-being requires to meet psychological needs as autonomy and competence. However, dehumanization decreases the ability to meet these needs, therefore it can directly contribute to workers’ dissatisfaction. Job satisfaction is part of the dignity of people at work, so violating this dignity would also imply violating the perception of job satisfaction (Bolton, 2010). In this regard, dehumanization would be a form of violation of the dignity of employees since these are treated to fulfil the achievements of the organization (Kaufmann et al., 2011). Furthermore, it has recently been found that there is a significant and negative effect between dehumanization and employee satisfaction (Caesens et al., 2017). Based on this rationale we stated the following hypothesis:

*H2:* The perception of organizational dehumanization is negatively related to the job satisfaction of workers.

### Organizational Dehumanization as a Mediating Variable Between Authentic Leadership and Job Satisfaction

As mentioned, authentic leadership helps workers perceive satisfaction in their workplace (Baek et al., 2019; Wong et al., 2020), increases their self-determination, fosters intrinsic motivation and a culture of openness at the organization, which allows creating a learning context and promotes conditions for the positive development of the followers (Gardner et al., 2005). Therefore, it is expected that the job satisfaction of workers is influenced by their perception of organizational dehumanization since dehumanizing experiences cause a decrease in job satisfaction of workers as it violates their basic needs (Gillet et al., 2012; Shore & Coyle-Shapiro, 2012). Similarly, studies of organizational dehumanization have found that mechanically dehumanizing experiences can have effects on the well-being of workers (Bastian & Haslam, 2011; Christoff, 2014). In this sense, Christoff (2014), in his study affirms that dehumanization in the organization can harm the well-being of workers since it increases the level of anxiety or depression of these and decreases the need for competence and interaction between workers. According to this point of view, Baldissarri et al. (2014), found that workers who felt perceived as an instrument by their supervisor reported higher levels of burnout.

**Figure 1:**
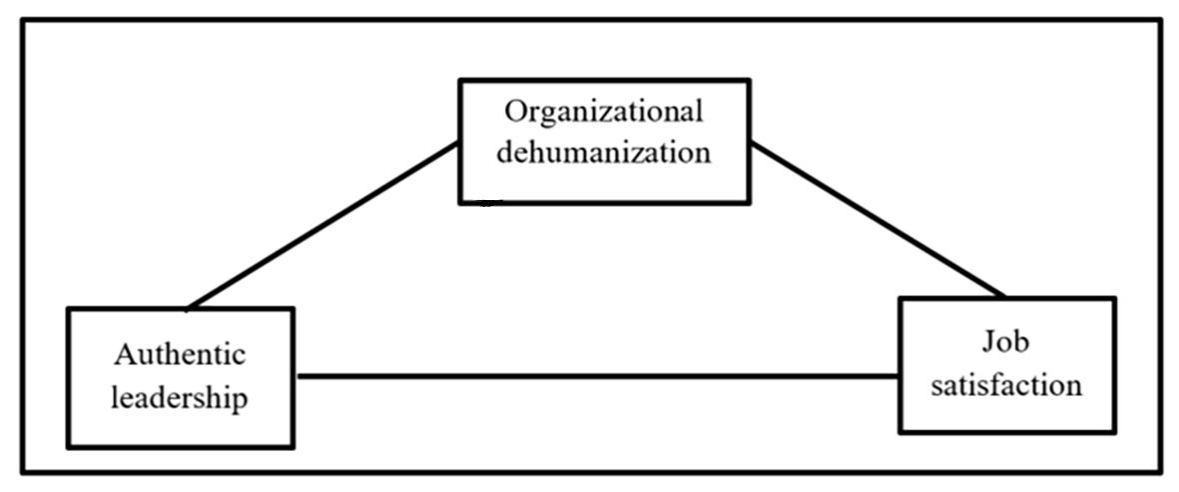
Proposed Model

As has been detailed bibliographically, the level of job satisfaction that workers perceive depends on the type of leadership experienced and the humanization or dehumanization that they perceive from their organization. So, the influence of leadership style on job satisfaction can be mitigated through the effect of organizational dehumanization. The proposed model is shown in Figure 1. Thus, it is proposed that:

*H3*: Organizational dehumanization mediates the relationship between authentic leadership and job satisfaction.

## Method

The study had a non-experimental quantitative cross-sectional approach (Kerlinger & Lee, 2002). The sample was comprised by 422 workers from for-profit or non-profit organizations, from different sectors, since there was a need to understand the subject in a wide variety of roles. The sample was chosen non-probabilistically, for convenience. In order to allow participants to be familiar with their organization, we use as an inclusion criterion to have a minimum stay of 6 months working for the organization. From participants, 50.7% (214) were men and 49.3% (208) women, with an average age of 38.96 years. They worked mainly for the Social Services, Health and Education sector with 25.1%, followed by the Commerce, Hotels and Restaurants sector with 17.8% of the total sample. Regarding the educational level, 41.3% had a university degree and 9.7% had postgraduate studies. 62% of the sample reported a full-time job, working 45 hours per week. The average time of tenure was 6.95 years (*SD* = 7.86).

The goal of this study is to analyze the relationship between authentic leadership and job satisfaction and the mediator role of organizational dehumanization.

### Instruments

#### Organizational Dehumanization

It was measured by the Organizational Dehumanization Scale of Caesens et al. 2017 adapted to the Spanish from Ariño-Mateo et al. (2021). It has one and 10 items answered on a Likert scale from 1 ("total disagreement") to 7 ("total agreement"), Item example is "*My organization would not hesitate to replace me if that allowed the company to obtain greater benefits.*" Cronbach's alpha in the current study was .923 and McDonald's omega was .924.

#### Authentic Leadership

It was measured by the scale of Authentic leadership created by Walumbwa et al. (2008) and validated by Moriano et al. (2011) The questionnaire is made up of four factors: transparency in relationships (3 items, item example: "*encourages each person to express their opinion*"), internalized morality (3 items, item example: "*asks you to assume positions that are in accordance with the values* ​​*that are important to you*”), balanced processing (3 items, item example: “*seeks the opinion of others to improve relationships with them”)* and self-awareness (4 items, item example: *“knows when it is time to re-examine your position on important issues”),* which in total add up to 13 items. The responses were on a Likert scale ranging from 0 ("never") to 5 ("always"). In the current study, Cronbach's alpha = .975 and McDonald's omega = .975.

#### Job Satisfaction

Meliá and Peiró S20/23 Job Satisfaction test was used to assess job satisfaction (Meliá and Peiró, 1989). It has five dimensions: (1) Satisfaction with supervision (item example, “*the way their supervisors judge their task*”); (2) Satisfaction with the physical environment, (item example, “*the physical environment and the space available to you in your workplace*”); (3) Satisfaction with the benefits received (item example, “*the promotion opportunities you have*”); (4) Intrinsic job satisfaction (item example, "*the satisfaction that your job produces for itself*"); and (5) Satisfaction with participation (item example, “*your participation in the decisions of your department or section*”). The instrument has a total of 23 items to be answered by a seven-point Likert response format (from Very Satisfied = 7 to Very Dissatisfied = 1). For the study sample, Cronbach's alpha = .962, and McDonald's omega = .963.

### Procedure

Participants were accessed by social networks sampling and snowball data collection methodology. They were invited either to answer an online questionnaire on the Survey Monkey platform or answer a paper-based survey questionnaire (where workers have the chance to give them back a week later).

All the respondents were explained about the objectives of the study, the confidentiality of their answers and the voluntary nature of their participation. Data were collected from March to June 2018. In total, more than 800 surveys were delivered in both formats, and 422 surveys were completed, with a response rate of 52.8%.

The questionnaire consisted of two sections. The first requested sociodemographic data and characteristics of the participants' workplace and the second one focused on questions from the three research constructs, organizational dehumanization, job satisfaction, and authentic leadership. The average time to answer was 25 minutes. The sample was obtained in four months.

### Analysis

Descriptive, correlational and mediation analyses were carried out. The elements described by Baron and Kenny (1986) were tested to evaluate the mediating effect of dehumanization between the other two variables: (1) a significant relationship between the independent variable and the mediator; (2) significance between the independent and the dependent variable; (3) importance between the mediator and the dependent variable; and (4) controlling the influence of the mediator, determining if the original relationship between the independent and dependent variables is reduced to non-importance or becomes smaller, which provides evidence of total or partial mediation. For this, the macro created by Hayes (2013) was used. IBM Statistical Package for the Social Sciences (SPSS 24.0) was used, and confirmatory factor analysis was performed using Mplus V.7 software.

## Results

A confirmatory factor analysis with the maximum likelihood method with oblique rotation was run for each instrument, maximum likelihood was used because the study variables have more than 5 categories, therefore they can be called continuous (Rhemtulla et al., 2012), in the case of oblique rotation, it is common for social sciences to use this form, due to that it is common for there to be a correlation between variables, instead varimax assumes that there is no correlation, using the Mplus V.7 software. Table 1 shows the results, indicating that each instrument is properly adjusted. In the case of the organization dehumanization scale, the indicators show: χ^2^ = 82.12, *df* = 32, χ^2^ / *df* = 2.57 (<3), SRMR = .06 (<.08), RMSEA = .09 (<.08), CFI = .93 (> .90) and TLI = .90; The authentic leadership adjusted: χ^2^ = 218.48; *df* = 98 and χ^2^ / *df* = 2.23; SRMR = .02; RMSEA = .05; CFI = .96; TLI = .96 and job satisfaction: χ^2^ = 610.38; *df* = 220 and χ^2^ / *df* = 2.77; SRMR = .05; RMSEA = .06; CFI = .92; TLI = .91. Values less than or equal to 0.09 are acceptable as long as RMSEA or CFI corroborates the model’s goodness of fit (Hu & Bentler, 1999); finally, values of CFI equal to 0.95 would indicate that a model shows good fit to the data (Hair et al., 2010; Hu & Bentler, 1999), though some authors maintain that values of .90 or even .80 are acceptable (Hair et al., 2010).

**Table 1:** CFA Results of the Scales Used in the Study

Variable	χ^2^	*df*	χ^2^/*df*	SRMR	RMSEA	CFI	TLI
Organizational dehumanization	82.12	32	2.57	0.06	0.09	0.93	0.90
Authentic leadership	218.48	98	2.23	0.02	0.05	0.96	0.96
Job satisfaction	610.38	220	2.77	0.05	0.06	0.92	0.91

*Note. N* = 422. SRMR = standardized root-mean-square residual; RMSEA = root-mean-square error of approximation; CFI = comparative fit index; TLI = Tucker-Lewis Index.

Results indicated a mean of 4.21 (*SD* = 1.49) on authentic leadership, as observed in Table 2. This scale has values ​​with a minimum of 0 and a maximum of 6. An average of 4.41 (*SD* = 1.39) on organizational dehumanization and 5.21 (*SD* = 1.18) on the job satisfaction scale was obtained, both scales having the same range of 1 as minimum and 7 as maximum.

**Table 2:** Descriptive Statistics of the Authentic Leadership, Organizational Dehumanization and Job Satisfaction Variables of the Total Sample

Variable	Minimum	Maximum	Mean	*SD*
Authentic leadership	0	6	4.21	1.49
Organizational dehumanization	1	7	4.41	1.39
Job satisfaction	1.13	7	5.21	1.18

*Note. N* = 422.

Authentic leadership is negatively correlated with organizational dehumanization (r = -0.284, *p* < 0.01) and positively with job satisfaction (r = 0.616, *p* < 0.01). Regarding the relationship of organizational dehumanization with the dependent variable, job satisfaction, it was found to have a negative and significant correlation with (r = -0.300, *p* < 0.01), (observed in Table 3). Thus, empirical support was obtained for Hypotheses 1 and 2, that is, authentic leadership is negatively related to the perception of organizational dehumanization (-0.295, *p* < 0.01) and perception of organizational dehumanization are negatively related to job satisfaction of workers (-0.104, *p* < 0.01) (see Table 4).

**Table 3:** Correlation Matrix Between the Study Variables

Variable	1	2	3
1. Authentic leadership	(.975; .975)	-0.284**	0.616**
2. Organizational dehumanization		(.923; .924)	-0.300**
3. Job satisfaction			(.962; .963)

*Note. N* = 422. Values in parentheses represent Cronbach’s alpha and McDonald's omega.

*p < .05. **p < .01.

**Table 4:** Results of Organizational Dehumanization as a Mediating Variable Between Authentic Leadership and Job Satisfaction

	Non-standardized coefficients				95% Bootstrapping CI	
Relation	Coefficient	Standard error	*t*	*p*	*LL*	*UL*
AL → ODH (a)	-0.295	0.051	-5.770	<.001	-0.396	-0.195
ODH → JS (b)	-0.104	0.032	-3.218	<.001	-0.167	-0.040
AL → ODH -> JS (c´)	0.458	0.035	13.280	<.001	0.391	0.526
AL → JS (c)	0.489	0.032	15.100	<.001	0.425	0.553
Indirect effect	0.031	0.011			0.013	0.055

*Note.* AL = Authentic leadership; ODH = Organizational dehumanization; JS = Job satisfaction; CI = confidence interval; *LL* = lower limit; *UL* = upper limit. a, b, c, c' = direct effects of the mediation analysis (see Figure 2).

Regarding criteria (1), (2) and (3) of mediation (Baron and Kenny, 1986), Table 4 shows that for all criteria there is a significant relationship between the variables. Furthermore, Table 4 shows a significant relationship between the study variables and a significant indirect effect of 0.031 given that 0 is not found in the 95% confidence intervals. Therefore, the organizational dehumanization variable works as a mediating variable in the relation previously exposed.

The indirect effect is 0.031, which means that two workers who differ in one point in the perception of authentic leadership, differ in their perception of job satisfaction by 0.031 units. This occurs because of the authentic leadership effect on organizational dehumanization, which in turn affects job satisfaction. This indirect effect is statistically different from zero, as revealed by a 95% boot confidence interval (0.013 to 0.055 as shown in Table 4 under the heading "95% Bootstrapping CI".

**Figure 2:**
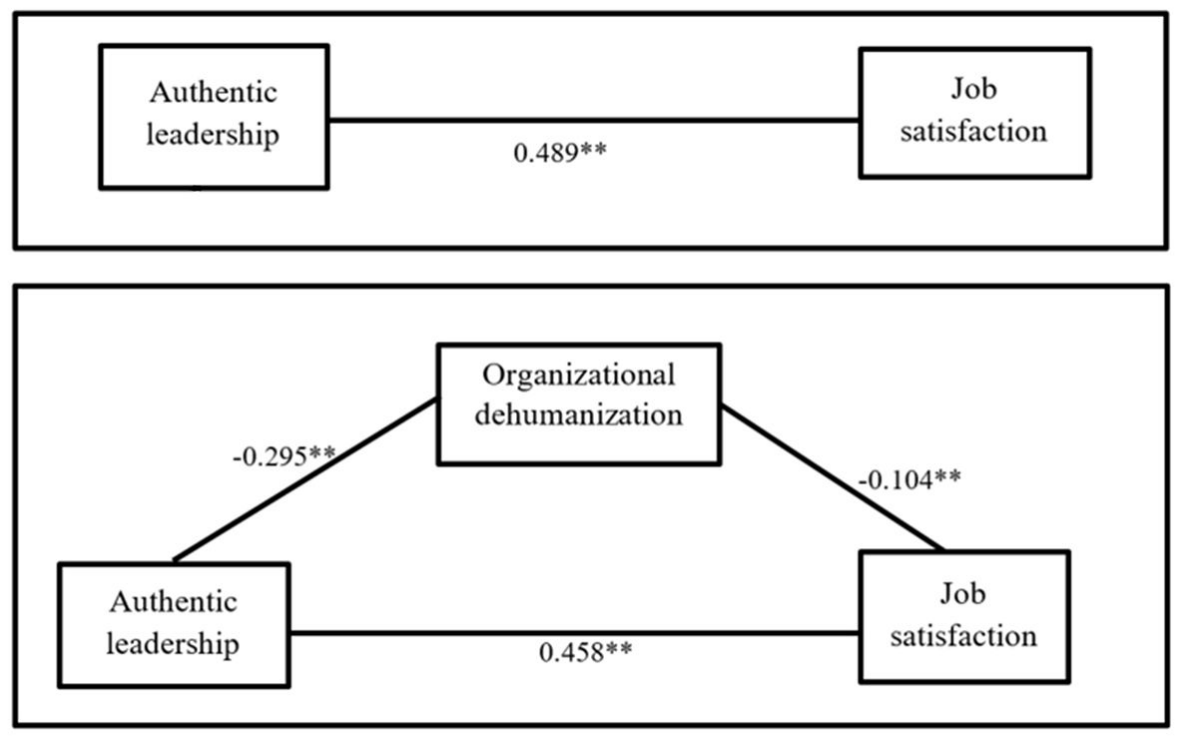
Mediation Model

Figure 2 shows that there is a significant relationship between the study variables. Furthermore, the total effect of authentic leadership on job satisfaction (0.489, p < 0.05) is greater than the direct effect (0.458, p < 0.05), therefore it is possible to indicate that organizational dehumanization mediates the relationship between authentic leadership and satisfaction labour.

In other words, organizational dehumanization is explaining the relationship between authentic leadership and job satisfaction. This allows us to maintain Hypothesis 3, organizational dehumanization mediates the relationship between the independent and dependent variable (see Figure 2).

## Discussion

The study allows identifying the relationships between authentic leadership, organizational dehumanization, and job satisfaction. First, leadership style influences both dehumanization and job satisfaction, and the research allowed obtaining empirical support for hypotheses about the mediating role of organizational dehumanization between the other two variables. Consequently, the effect of authentic leadership on job satisfaction is partially explained by organizational dehumanization. Furthermore, workers may perceive dehumanization by their organization depending on their leader, and this fact will have an impact in their job satisfaction.

Authentic leadership was found to be positively related to the job satisfaction of workers, as several studies have already stated (Giallonardo et al., 2010; Khan et al., 2017; Walumbwa et al., 2008). According to Ilies et al. (2005), authentic leaders positively influence the behaviour of workers as they provide support for the self-determination of followers. Gardner et al. (2005) indicate that internal regulation, characteristic of authentic leaders, contributes to higher levels of well-being, commitment and performance of employees. In conclusion, authentic leaders, (that is a self-conscious leader, which means that processes the information and handles it in a balanced way), presents an internalized moral perspective, they are transparent in their relationships, which is positively related to workers’ satisfaction.

Results allow confirming the second hypothesis, that is perception of organizational dehumanization is negatively related to job satisfaction. This is consistent with the study by Caesens et al. (2017), that state that an organization that does not meet the psychological needs of autonomy and competence, as the case of workers that perceive dehumanization, will show lower satisfaction.

On the other hand, as various studies indicate, it is not possible to ignore the context in leadership studies (Avolio et al., 2004; Day & O’Connor, 2003), in our study organizational dehumanization was identified as a mediating variable, that is, provides background on how authentic leadership affects job satisfaction.

In general, these findings are consistent with the proposal of Väyrynen and Laari-Salmela (2018) that organizational benevolence, or diminished dignity, should be related to the worker's perception of organizational dehumanization. Dehumanizing experiences promote a decrease in job satisfaction because they violate the basic needs of workers (Gillet et al., 2012; Shore & Coyle-Shapiro, 2012). This fact is important because the effect of authentic leadership on job satisfaction is partially explained through dehumanization.

These results allow us to better understand the role that organizational dehumanization plays with different variables of work and organizational psychology, in this case, authentic leadership and job satisfaction. The most important implications of this research are that it demonstrates the importance of organizational dehumanization over other processes and especially its mediating power. Results show the negative consequences of dehumanization despite having leaders who are authentic, and how job satisfaction can be overshadowed by the presence of organizational dehumanization. Therefore, although the worker is influenced by the positive attributes of an authentic leader, these can be minimized by negative perceptions that come from the organization, it is necessary not only to worry about the attributes of the leaders and how to find those that have characteristics of authentic but also, promote organizations where the worker perceives treated as a person with their attributes and capabilities and not as an object or number for the results of the company.

Finally, this study provides a unique perspective when analyzing authentic leadership and the perception of workers in their organization, and how these variables can increase the perception of organizational dehumanization in public and private organizations in Chile.

Despite the study's contributions, there are limitations that need to be noted. Firstly, this research was cross-sectional, therefore, to avoid the uncertainty of a causal relationship, future research may apply a longitudinal study design to this studied model. Therefore, it is recommended to use experimental (randomizing participants to analysis groups) or longitudinal (Berlinger et al., 1988; Parker & Turner, 2002) research designs.

Second, even though worker participation was ensured to be confidential and anonymous, the data may be biased by social desirability. On the other hand, it would be interesting to delve into finding the factors that trigger the perception of organizational dehumanization in workers, either in the type of leadership in the design of the job or in the organizational goals. The type of organizational dehumanization seen in the study corresponds to the mechanistic dehumanization nature, but it is possible that in organizational contexts there is also animalistic dehumanization.

Finally, future researchers are left to carry out studies aimed at understanding the relationship between organizational dehumanization and mental disorders such as depression and anxiety. In addition, it is necessary to analyze which of the four dimensions of authentic leadership has a greater preponderance when we talk about its relationship with authentic leadership.

Regarding the practical implications, the evidence provided by this research confirms that those workers who have an authentic leader will present greater job satisfaction, and furthermore, that this relationship may be affected by organizational dehumanization. Therefore, it is important that when selecting the formal leaders of an organization, they have attitudes such as an internalized moral perspective, the ability to process information objectively and make decisions according to the well-being of the workers and organization. This fact is of great relevance since when workers show high levels of job satisfaction, their absenteeism is reduced, staff retention increases, improving the organization´s productivity due to the related costs lost (Mendoza-Llanos, 2015). For this reason, it is necessary for managers to pay attention to the personnel selection processes of the company's management team. (Furthermore, investigating the characteristics of authentic leadership mentioned previously, and on reducing or eliminating the perception of dehumanization in the work environment). In addition, organizations have a tool with which they can examine the level of dehumanization of workers and invest in resources that can attenuate or diminish the perception of dehumanization.

Likewise, this study presents the possible negative implications that dehumanization can generate. At the individual level, dehumanization increases “cognitive deconstructive” states characterized by decreased clarity of thought, emotional exhaustion, lack of cognitive flexibility, and an absence of meaningful thinking (Bastian & Haslam, 2011; Twenge et al., 2003).

## Conclusions

This study investigates organizational dehumanization in Latin American contexts. Organizational dehumanization was found to have a negative relationship with job satisfaction and authentic leadership. Furthermore, this research shows the mediating role of organizational dehumanization in the relationship between authentic leadership and job satisfaction. Consequently, employees who have a genuine leader but who work in an environment that they perceive as dehumanizing will have less job satisfaction, and this will consequently affect the overall organization performance.

These findings, further highlight the role that organizational dehumanization can play, should alert organizations to understand, analyze and, if necessary, improve the perception of organizational dehumanization to help mitigate the effects that organizational dehumanization can have at the level of people's well-being and their working environment.

## Data Availability

The data that support the findings of this study are available from the corresponding author upon reasonable request.
